# The African wildlife ontology tutorial ontologies

**DOI:** 10.1186/s13326-020-00224-y

**Published:** 2020-06-23

**Authors:** C. Maria Keet

**Affiliations:** Department of Computer Science, University of Cape Town, 18 University Avenue, Rondebosch, Cape Town, South Africa

**Keywords:** Ontology engineering, Tutorial ontology, African wildlife

## Abstract

**Background:**

Most tutorial ontologies focus on illustrating one aspect of ontology development, notably language features and automated reasoners, but ignore ontology development factors, such as emergent modelling guidelines and ontological principles. Yet, novices replicate examples from the exercise they carry out. Not providing good examples holistically causes the propagation of sub-optimal ontology development, which may negatively affect the quality of a real domain ontology.

**Results:**

We identified 22 requirements that a good tutorial ontology should satisfy regarding subject domain, logics and reasoning, and engineering aspects. We developed a set of ontologies about African Wildlife to serve as tutorial ontologies. A majority of the requirements have been met with the set of African Wildlife Ontology tutorial ontologies, which are introduced in this paper. The African Wildlife Ontology is mature and has been used yearly in an ontology engineering course or tutorial since 2010 and is included in a recent ontology engineering textbook with relevant examples and exercises.

**Conclusion:**

The African Wildlife Ontology provides a wide range of options concerning examples and exercises for ontology engineering well beyond illustrating just language features and automated reasoning. It assists in demonstrating tasks concerning ontology quality, such as alignment to a foundational ontology and satisfying competency questions, versioning, and multilingual ontologies.

## Background

The amount of educational material to learn about ontologies is increasing gradually, and there is material for different target audiences, including domain experts, applied philosophers, computer scientists and software developers, and practitioners. These materials may include a tutorial ontology to illustrate concepts and principles and may be used for exercises. There are no guidelines as to what such a tutorial ontology should be about and should look like. The two most popular tutorial ontologies are about wine and pizza, which are not ideal introductory subject domains on closer inspection (discussed below), they are limited to OWL DL only, and are over 15 years old by now, hence, neither taking into consideration the more recent insights in ontology engineering nor the OWL 2 standard with its additional features.

Considering subject domains in the most closely related area, conceptual modelling for relational databases, there is a small set of universes of discourse that are used in teaching throughout the plethora of teaching materials available: the video/DVD/book rentals, employees at a company, a university, and, to a lesser extent, flights and airplanes. Neither of these topics for databases lend themselves well for ontologies, for the simple reason that the two have different purposes. It does raise the question as to what would be suitable and, more fundamentally, what it is that makes some subject domain suitable but not another, and, underlying that, what the requirements are for an ontology to be a good tutorial ontology.

In this paper, we will first analyse existing tutorial ontologies and highlight some issues. We then proceed to formulate a preliminary, first, list of requirements that tutorial ontologies should meet. The African Wildlife Ontology (AWO) tutorial ontologies are then introduced briefly and held against the requirements. The scope of this paper is thus to introduce the AWO tutorial ontologies and to frame it in that context. Finally, we discuss and conclude.

### Tutorial ontologies: issues and comparison

There are several tutorial ontologies, which are summarised in Table 1 and discussed in this subsection; the next subsection that summarises the problems.

**Table 1:** Summary of main extant tutorial ontologies

Ontology	Year	Stated aim	Content	Language	Modelling issues	Automated reasoning	Current OE (e.g., ODP, FO)
wine	2001	novice ‘all aspects’ (methodology, modelling, reasoning) for OE	somewhat generic, repetitive, limited extensibility	frames; the wine.owl in OWL DL is based on it	multiple (e.g., class vs instance, hasX)	yes	no
pizza	2004	Protégé user guide, also illustrate OWL and reasoning	somewhat generic, repetitive, limited extensibility	OWL DL	multiple (e.g., hasX, FO commitment, lack of domain & range)	yes	no
university	2005	illustrate OWL and reasoning	generic, CDM (cf. ontology) scope, very small	<OWL DL (*ALCIN*)	multiple (e.g., XorY, naming of individuals)	yes	no
zooAnimals	2011	illustrate DL&OWL and some GoodOD modelling guidelines	generic, a lot of detail, easily extensible	<OWL 2 DL (*SHO*)	few	yes	partially (BioTopLite)
family history	2013	illustrate OWL 2 DL and reasoning	specific to author’s family, not extensible	OWL 2 DL	multiple (e.g., hasX, FO commitment, lack of domain & range)	error	no
shirt	2015	illustrate the design of the FMA	generic, structure specific to FMA, repetitive, not extensible	<OWL 2 DL (*ALCIQ*)	few (lack of domain & range)	none	very limited (reference ontology)

Abbreviations: OE: ontology engineering; ODP: ontology design pattern; FO: foundational ontology; CDM: conceptual data model; FMA: foundational model of anatomy; OWL DL is *SHOIN*(*D*) and OWL 2 DL is *SROIQ*(*D*) in DL notation

Of the six tutorial ontologies considered in detail, two are popular, being the Wine Ontology and the Pizza ontology, since they are part of the W3C OWL guide and designed for the most popular ontology development environment (Protégé), respectively. They have various shortcomings as tutorial ontologies, however, especially concerning modelling practices or styles (see also [1]).

The Wine ontology in its current form emanates from the “Ontology development 101” tutorial [2] with its frames and slots that was subsequently transferred into OWL^1^ and used in the “OWL guide” [3], which is a W3C Recommendation. While the guide contains some good suggestions, such as that “Synonyms for the same concept do not represent different classes” [2], there are also modelling issues, notably that the ontology is replete with the class-as-instance error that is promoted by the incorrect statement in the tutorial “Individual instances are the most specific concepts represented in a knowledge base.” [2] (e.g., TaylorPort as instance of Port and MalbecGrape as instance of Grape instead of as subclass of), and the sub-optimal object property naming scheme of ‘hasX’, such as adjacentRegion between two Regions rather than the reusable and generic adjacent. Further, it uses different desiderata in the direct subclassing of wine such as the likes of Bordeaux and Loire (region-based) and Chardonnay and Cabernet Sauvignon (grape-based), and then there are other criteria, like DessertWine (food pairing-based grouping) and ‘wine descriptor’ ones (DryWine, RedWine, TableWine), This does make it interesting for showing classification reasoning (except the undesirable deduction that DryWine ≡ TableWine), but is not ideal from a modelling viewpoint. Further, from a tutorial viewpoint: there are many repetitions, such as very many wineries, which distract from the principles, and it lacks annotations.

The Pizza ontology tutorial was created for the Protégé user manual and OWL DL ontology language [4]. It reflects the state of the art at that time, yet much has happened over the past 15 years. For instance, there are new OWL 2 features and there are foundational ontologies that provide guidance for representing attributes (cf. Pizza’s ValuePartition). Pizza’s DomainConcept throws a learner straight into philosophical debates, which may not be useful to start with, and, for all practical purposes, duplicates owl:Thing. Like the Wine ontology, it has the ‘hasX’ naming scheme for object properties, such as hasTopping, including the name of the class it is supposed to relate to, which is a quirk that is a combination of a workaround for not having qualified number restrictions (an OWL 1 artefact) and of a sub-optimal ontological analysis of the relation (*in casu*, of how the toppings really relate to the rest of the pizza) that reduces chance of ontology reuse and alignment. Also, this propagates into students’ modelling approaches: students’ ontologies in earlier instances of the author’s course on ontology engineering included, among others, a sandwich ontology with hasFilling, an electrical circuit board ontology with hasIsolator, furniture with hasHeadboard. Modelling issues are compounded by the statement “we generally advise *against* doing [domain and range declarations]” in the tutorial documentation. When one aims to get novices to use Protégé and OWL so as not get too many error with the automated reasoners, that might make sense, but ontologically, fewer constraints make an ontology less good because it admits more unintended models. Finally, it has repetitive content to show features, which may be distracting, and, as with Wine, there is only one ‘final’ ontology, despite that multiple releases are common in practice.

Other tutorial ontologies include Family History, zooAnimals, University, and Shirt. Family History [5] is developed by the same group as Pizza and aims to teach about advanced OWL 2 features and maximise the use of inferencing. Loading it in Protégé 5.2 results in three punning errors, since it mixes three object properties with annotation properties (affecting 32 axioms), which is disallowed, and trying to classify it without the three annotation properties returned an OutOfMemoryError (on a MacBookPro, 2.6 GHz and 8GB of memory), which is not ideal to start a tutorial with. Concerning modelling issues, ParentOfRobert illustrates one can use individuals in class expressions, but just that the language allows it, does not mean it is ontologically a good idea that must be taught. It also has the ‘hasX’ semantic weakness, very few annotations, DomainEntity being subsumed by owl:Thing, and multiple data properties. In contrast to Pizza and Wine, all the declared instances are instances and the ontology has different versions as one goes along in the chapters. It has some subject domain aspects descending into politics, which would render it unsuitable for teaching in several countries, such as stating that Sex ≡ Female ⊔ Male (enforcing a gender binary) and that Person$\sqsubseteq \leq 2$hasParent.Person (multiple constructions are possible biologically, societally, and legally).

The remaining tutorial ontologies have been developed by different ‘schools’ of views on ontology engineering (OE), which is readily apparent in their scope and content. The zooAnimals tutorial ontology [6] comes closest to our aims for a versatile tutorial ontology, demonstrating multiple OWL features, avoiding modelling issues such as class vs instance, and it is informed by a top-domain ontology (BioTop) as well as deep philosophical notions such as dispositions. It puts them all together into one ontology instead of gradual extensions, however, which is off-putting at a novice stage. One may quibble about some of the content, such as simplifications that Plant≡∃hasProperPart.Chloroplast (notably, some parasitic plants and all myco-heterotrophic plants do not have chloroplasts) and there are unintended undesirable deductions—i.e., logically implied, but incorrect ontologically—such as marineAnimal$\sqsubseteq $Omnivore since not all such animals are omnivores. Any simplified ‘common generic subject domain’ is likely to have some shortcuts that are not 100% scientifically accurate, and it may be a fine line between tutorial approximation and modelling mistake.

The University ontology focuses on illustrating OWL features and automated reasoning, rather than modelling. For instance, it has AcademicStaff with sibling NonAcademicStaff where a “non-X” complement class is sub-optimal, especially when there is a term for it. The representation of Student$\sqsubseteq $Person is an advanced modelling aspect that can be improved upon with a separate branch for roles played by an object. The Computer Science Ontology was based on the University Ontology tutorial and contains artificial classes, like unions of classes (ProfessorinHCIorAI) and underspecified or incorrect individuals like AI and HCI (e.g., some course instance would be CS_AI-sem1-2018 instead).

The Shirt ontology is a tutorial ontology to explain the structure and organisation of the Foundational Model of Anatomy in a simpler way^2^ and therefore does not have the hasX naming scheme for object properties, it has no data properties and no instances. It has many annotations with explanations of the entities. There are no inferences.

Regarding suitability of the subject domains of the ontologies assessed, they are mixed. Wine misses many wine-producing regions in the Americas (e.g., Chile), in Europe (e.g. Spain, Bulgaria), and elsewhere (e.g., South Africa) and Pizza lacks varieties beyond Italian and American ones, and both are served regularly in a relatively small part of the world, therewith reducing their appeal internationally. Family history and a university as subject domains veer too easily into the area of database design for a single application, rather than application-independent generic knowledge for an ontology. Shirts and zoo animals do not have these shortcomings.

Finally, more or less related textbooks were considered [7–11]. Only the “Semantic Web for the working Ontologist” (2nd ed.) has sample files for the book’s many small examples^3^ with two reoccurring subject domains, being English literature and products.

### Problems to address

The previous section described several problems with existing tutorial ontologies. Notably, the recurring shortcomings are that

i) good modelling practices are mostly ignored in favour of demonstrating language features, automated reasoning, and tools
ii) when good modelling practices and at least some recent ontology engineering advances are included, it falls short on language features and gradual extensions.

This has a negative effect on learning about ontology development, for tutorial ontology practices are nonetheless seen by students as so-called ‘model answers’ even if it were not intended to have that function.

The ontology survey does not reveal what may be the characteristics of a good tutorial ontology and, to the best of our knowledge, there is no such list of comprehensive criteria for tutorial ontologies specifically. Schober et al. [6] propose a partial list with seven high-level content requirements indeed, such as a common sense knowledge subject domain that lends itself well to demonstrate the “classic” modelling challenges, but it omits the essential components of logic, reasoning, and engineering requirements. Scoping it more broadly, one could consider modelling guidelines and automated checkers for production level ontologies, such as [12–15]. They can inform the development of tutorial ontologies, in particular to avoid such issues as the class vs. instance error in the provided sample ontology, but that is different from educating students about the foundations and reasons for such guidelines starting from a basic level of modelling to more advanced issues. For instance, disjointness and covering constraints among subclasses of a parent class is indeed desirable together with coherent criteria to declare a taxonomy [15], but that does not let students observe or experience mistakes, i.e., learn what is suboptimal or does not work and why. A tutorial ontology also would have to be able to accommodate common pitfalls and gradual quality improvements, among other things, which are not covered by the general guidelines. Also, general guidelines tend to follow one commitment over another—e.g., the GoodOD guidelines favour a realist approach with the BFO foundational ontology—but for teaching OE in general, students need learn to be cognisant of multiple possible commitments, their consequences when choosing one or the other, and have at least one practical example of such a difference to illustrate it, which general guidelines do not provide.

### Potential benefits of the African wildlife ontology tutorial ontologies

In order to address these problems, we introduce the African Wildlife Ontology (AWO). The AWO has been developed and extended over 8 years. It meets a range of different tutorial ontology requirements, notably regarding subject domain, use of language features and automated reasoning, and its link with foundational ontologies on the one hand and engineering on the other. It aims to take a principled approach to tutorial ontology development, which thereby not only may assist a learner, but, moreover from a scientific viewpoint, it might serve as a starting point for tutorial ontology creation or improvement more broadly, and therewith in the future contribute to an experimental analysis of tutorial ontology quality. This could benefit educational material for ontology development.

Also, educationally, there is some benefit to ‘reusing’ the same ontology to illustrate a range of aspects, rather than introducing many small ad hoc examples, for then later in a course, it makes it easier for the learners to see the advances they have made. This is also illustrated with offering multiple versions of the ontology, which clearly indicate different types of increments.

Finally, the AWO can be used on its own or together with the textbook “An Introduction to Ontology Engineering” [16], which contains examples, tasks and exercises with the AWO.

## Construction and content

The construction of the AWO tutorial ontologies has gone through an iterative development process since 2010. This involved various extensions and improvements by design, mainly to address the increasing amount of requirements to meet, and maintenance issues, such as resolving link rot of an imported ontology. Rather than describing the process of the iterative development cycles, we present here a ‘digest’ version of it. First, a set of tutorial ontology requirements are presented together, then a brief overview of the AWO content is described, and subsequently we turn to which of these requirements are met by the AWO.

### OE tutorial ontology requirements

Tutorials on ontologies may have different foci and it is unlikely that an ontology used for a specific tutorial will meet all requirements. The ontology should meet the needs for that tutorial or course, and that should be stated clearly. As such, this list is intended to serve as a set of considerations when developing a tutorial ontology. Each item easily can take up a paragraph of explanation. We refrain from this by assuming the reader of this paper is sufficiently well-versed in ontology engineering and seeking information on tutorial ontologies. For indicative purpose, the requirements are categorised under three dimensions: the subject domain of the ontology, logics & reasoning, and engineering factors.

#### Subject domain

The tutorial ontology’s subject domain, also called universe of discourse, should be versatile to be able to cater plausibly for a range of modelling aspects. We specify seven requirements for it, as follows.

1. It should be general and commonsensical domain knowledge, so as to be sufficiently intuitive for non-experts to be able to understand content and add knowledge. Optionally, it may be an enjoyable subject domain to make it look more interesting and, perhaps, also uncontroversial^4^ to increase chance of use across different settings and cultures.
2. The content should be not wrong ontologically, neither regarding how things are represented (e.g., no classes as instances) nor the subject domain semantics (e.g., whales are mammals, not fish).
3. It needs to be sufficiently international or cross-cultural so that experimentation with a scenario with multiple natural languages for multilingual ontologies is plausible.
4. Its contents should demonstrate diverse aspects succinctly when illustrating a point (in contrast to being repetitive in content).
5. It needs to be sufficiently versatile to illustrate the multiple aspects in ontology development (see below), including the use of core relations (e.g., mereology).
6. It should permit extension to knowledge that requires features beyond Description Logics-based OWL species, so as to demonstrate representation limitations and pointers to possible directions of solutions (e.g., temporal aspects, non-monotonicity, full first-order logic).
7. The subject domain should be able to possibly be used in a range of use case scenarios (database integration, science, NLP, and so on).

#### Logics & reasoning

Since a core feature of ontologies is their logic underpinning, a tutorial ontology thus also will need to meet criteria for the representation language and automated reasoning over it. They are as follows.

I. The ontology should be represented in a logic that has tool support for modelling and automated reasoning.
II. The ontology should be represented in a logic that has tool support for ‘debugging’ features that ‘explain’ the deductions, meaning at least showing the subset of axioms involved in a deduction.
III. It should permit simple classification examples and easy examples for showing unsatisfiability and inconsistency, such that it does not involve more than 2-3 axioms in the explanation, and also longer ones for an intermediate level.
IV. The standard reasoning tasks should terminate fairly fast (<5 s) for most basic exercises with the ontology, with the ‘standard’ reasoning tasks being subsumption/classification, satisfiability, consistency, querying and instance retrieval.
V. The representation language should offer some way of importing or linking ontologies into a network of ontologies.
VI. The language should be expressive enough to demonstrate advanced modelling features (e.g., irreflexivity and role composition).
VII. The logic should be intuitive for the modelling examples at least at the start; e.g., if there is a need for ternary relations, then the logic should permit *n*-aries with *n*≥3 so that it can be represented as such, rather than as an approximation with a reification and a workaround pattern.

#### Engineering and development tasks

An ontology is an artefact, which has to be built and maintained. To this end, there are multiple approaches, methodologies, methods, and software tools of which at least a subset will have to become part of an ontologist’s toolbox. We identified eight broad requirements:

A. At least some ontology development methods and tools should be able to use the ontology, be used for improvement of the ontology, etc.
B. The ontology needs to permit short/simple competency questions (CQs) and may permit long and complicated CQs, which are formulated for the ontology’s content and where some can be answered on the ontology and others cannot.
C. At least some of the top-level classes in the hierarchy should be straight-forward enough to be easily linked to a leaf category from a foundational ontology (e.g., Animal is clearly a physical object, but the ontological status of Algorithm is not immediately obvious).
D. It should be relatable to, or usable with, or else at least amenable to the use of, ontology design patterns, be they content patterns or other types.
E. It is beneficial if there is at least one ontology sufficiently related to its contents, so that it can be used for tasks such as comparison, alignment, and ontology imports.
F. It is beneficial if there are relevant related non-ontological resources that could be used for bottom-up ontology development.
G. It should be able to show ontology quality improvements gradually, stored in different files.
H. It should not violate basic ontology design principles (e.g., classes and relations vs. implementation decisions with data properties and data types when representing qualities of entities, such as an animal’s weight).

While this list may turn out not to be exhaustive in the near future, it is expected to be sufficient for introductory levels of ontology development tutorials and courses. Either way, this list of requirements are already hard to meet in one single ontology. For instance, simplicity (Items 3, III, and B) vs. complicated extensions and ontological precision (Items 6 and C) cannot be fully met at once. On the flip side, some requirements are closely related or overlap, such as design principles (Item H) and not being wrong ontologically (Item 2) since some of the former are informed by the latter.

### Content of the AWO – at a glance

**Fig. 1:**
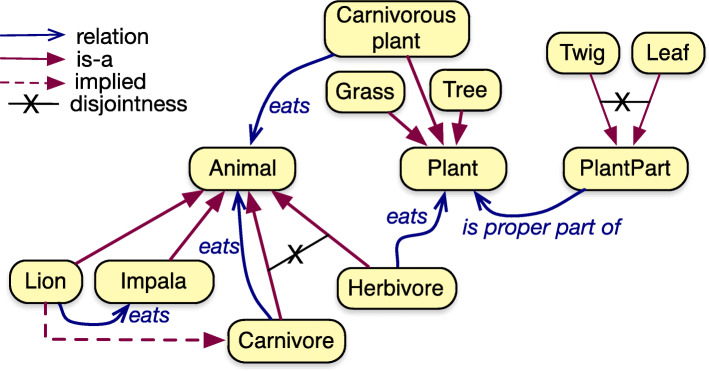
The African Wildlife Ontology at a glance. The main classes and relations of the African Wildlife ontology (v1) and an illustrative selection of its subclasses. (The relations drawn are existentially quantified.)

The principal content of the AWO is, in the first stage at least, ‘intuitive’ knowledge about African wildlife. This subject domain originated from an early Semantic Web book ([8], Section 4.3.1) that was restructured and extended slightly for its first, basic version; see Table 2 and Fig. 1. It has descriptions of typical wildlife animals, such as Lion and Elephant, and what they eat, including Impala (a type of antelope), and Twig or Leaf, respectively. Basic extensions in the simple version of the ontology (v1) include plant parts, so as to demonstrate parthood and its transitivity, and carnivore vs. herbivore, which make it easy to illustrate disjointness, subsumption reasoning, and unsatisfiable classes, and carnivorous plants to demonstrate logical consequences of declaring domain and range axioms 5. Most elements have been annotated with informal descriptions, and several annotations link to descriptions on Wikipedia.

**Table 2:** AWO ontologies, with their main differences

File name	Difference
AfricanWildlifeOntology.xml	This is the file from http://www.iro.umontreal.ca/~lapalme/ift6281/OWL/AfricanWildlifeOntology.xml, that was based on the description in [8]
AfricanWildlifeOntologyWeb.owl	AfricanWildlifeOntology.xml + changed the extension to .owl and appended the name with Web. This ontology gave at the time (in 2010) a load error in the then current version of Protégé due to the use of Collection in the definition of Herbivore
AfricanWildlifeOntology0.owl	AfricanWildlifeOntologyWeb.owl + that section on Collection removed
AfricanWildlifeOntology1.owl	AfricanWildlifeOntology0.owl + several classes and object properties were added (up to *SRI* DL expressiveness), more annotations, URI updated (described in Example 4.1 in [16])
AfricanWildlifeOntology1a.owl	AfricanWildlifeOntology1.owl + new content for a selection of the CQs in Exercise 5.1 in [16] (its CQ5, CQ8) and awo_12 of the CQ dataset [18])
AfricanWildlifeOntology2.owl	AfricanWildlifeOntology1.owl + OWL-ised DOLCE (Dolce-Lite.owl) was imported and aligned
AfricanWildlifeOntology2a.owl	AfricanWildlifeOntology2.owl + answers to the questions in Example 6.2 in [16] on foundational ontology alignment
AfricanWildlifeOntology3.owl	AfricanWildlifeOntology1.owl + BFO v1 was imported and aligned
AfricanWildlifeOntology3a.owl	AfricanWildlifeOntology3.owl + answers to the questions in Example 6.2 in [16] on foundational ontology alignment
AfricanWildlifeOntology3b.owl	AfricanWildlifeOntology1.owl + BFO v2 + answer to Exercise 6.7 in v2 of [16] on refactoring the AWO with dispositions
AfricanWildlifeOntology4.owl	AfricanWildlifeOntology1.owl + some things cleaned up (e.g., consistent naming) and added some science content, more OWL language features are used (up to *SRIQ*), and several educational explanations and questions for further exploration have been added in annotation fields
AfricanWildlifeOntologyZU.owl	Mostly AfricanWildlifeOntology1.owl but then in isiZulu, with IRI changed
AfricanWildlifeOntologyAF.owl	AfricanWildlifeOntology1.owl but then in Afrikaans, has some IRI issues to resolve
AfricanWildlifeOntologyNL.owl	AfricanWildlifeOntology1.owl in Dutch, with IRI changed
AfricanWildlifeOntologyES.owl	AfricanWildlifeOntology1.owl in Spanish, same IRI but different file name

Like the aforementioned Family History ontology, there are several versions of the AWO that reflect different stages of learning. In the case of the AWO, this is not specifically with respect to OWL language features, but one of notions of ontology quality and where one is in the learning process. For instance, version 1a contains answers to several competency questions—i.e., quality requirements that an ontology ought to meet [17]—that were formulated for Exercise 5.1 in the “Methods and methodologies” chapter of [16]. Versions 2 and 3, on the other hand, have the AWO aligned to the DOLCE and BFO foundational ontologies, respectively, whose differences and merits are discussed in Chapter 6 of the textbook, ensuring discussion of refinements in ontological precision with, e.g., *processes* and *dispositions* (e.g., an Eating class with participating objects cf. an eats object property). Their respective versions with the answers to the related exercises have the name appended with an ‘a’ as well. Version 4 has some contents ‘cleaned up’, partially based on what the OOPS! tool [14] detected; it uses more advanced language features; and takes steps in the direction of adhering to science more precisely with finer granularity, such as type of carnivores and distinguishing between types of roots.

There are also four versions in different natural languages, being in isiZulu, Afrikaans, Dutch, and Spanish, which mainly serve the purpose of illustrating issues with multilingual settings of ontology use, which relates to content in Chapter 9 of the textbook.

### AWO against the requirements

The AWO meets most of the requirements (see Table 3). Concerning the subject domain, the content is general, versatile, not wrong, sufficiently international, and not repetitive (Items 1-4). The AWO includes the core relation of parthood for, especially, plants and their parts, with optional straightforward extensions with the participation relation (e.g., animals participating in a Chasing event) and membership (animal collectives, such as Herd; see v4 of the AWO), therewith meeting Item 5. Representation of relevant domain knowledge beyond Description Logics-based OWL species (Item 6) could include information about temporal segregation of foraging or commensalism, inclusion of species with distinct successive phases with substantial morphological changes (e.g., Caterpillar/Butterfly), and the notion of rigidity between what an object is and the role it plays (e.g., Lion playing the role of Predator; see v4 of the AWO). The subject domain is also fertile ground for exceptions that may be represented with non-monotonic logics; typical examples are that, generally, birds fly and plants have chlorophyl, but not all of them (e.g., the penguin and the dodder, respectively). Use case scenarios (Item 7) may be, among others, science of African wildlife, activism on endangered species, and applications such as a database integration and management system for zoos and for tourism websites.

**Table 3:** Summary of the evaluation of the AWO against the requirements

Item	Eval.	Item	Eval.	Item	Eval.
1	+	I	+	A	+
2	+	II	+	B	+
3	+	III	+	C	+
4	+	IV	+	D	±
5	+	V	+	E	–
6	±	VI	+	F	±
7	+	VII	–	G	+
				H	+

The evaluation (Eval.) can be either + fully met, ± partially met (not implemented), or – not met

Regarding the logics and reasoning, the AWO is represented in OWL [19], and thus has ample tooling support for knowledge representation, reasoning, and basic debugging/explanation, with ontology development environment tools such as Protégé (Items I-III). The AWO has both ‘simple’ deductions and more elaborate ones (Item III); e.g., compare Lion that is classified as a Carnivore, having one explanation involving three axioms, with Warthog that is classified as an Omnivore, for which there are three justifications computed that each use, on average, five axioms. Because the AWO is small, does not make extensive use of individuals and high number restrictions, the reasoner terminates fast under all standard reasoning tasks (Item IV). OWL has the language feature to import other ontologies and it also can be used in other ontology network frameworks, notably the Distributed Ontology, Model, and Specification Language DOL [20] (Item V). While OWL contains expressive features such as role chaining (Item VI), it, arguably, fails on intuitiveness especially for novices (Item VII). Regarding the latter, e.g., for as of yet unclear reasons, novices make errors in the use of existential and universal quantification [4, 13, 14], which is not known to be a problem as such when modelling the equivalent in, say, UML Class Diagrams, and there is the elaborate *n*-ary (with *n*≥3) approximation issue.

With respect to the engineering aspects, by virtue of the AWO being represented in OWL, there are tools that can process the ontology (Item A), and therewith ontology quality improvement methods can be used with the AWO. They include, e.g., the popular Protégé, and various tools for methods and quality, such as test-driven development [21] and OOPS! [14], and ontology development support activities, such as visualisation and documentation (e.g., [22, 23]). There are also a few competency questions that can be answered and that can be easily modelled to be answered, as included in AWO version 1a (Item B), and there are examples and activities to link it to foundational ontologies (AWO versions 2 and 3) with easy examples (Item C) (see below, ‘Utility and Discussion’). There are several versions demonstrating various quality improvements (Item G), avoiding violating some basic design principles like data properties and punning hacks (Item H), and touching upon some advanced engineering issues with multilingual ontologies (see Table 2).

It falls short at the novice level on two requirements: an easy way to link it to another ontology (Item E) and bottom-up development from non-ontological resources (Item F). It is possible and feasible in a mini-project assignment, however; e.g., one could use the freely available wildlife trade data^6^ or relate it to the Biodiversity Information Standards^7^ for application scenarios, and link it to the Envo Environment ontology [24] or take it easier on the domain knowledge with one of the available tourism ontologies to create an ontology network. A bottom-up approach to knowledge acquisition for ontologies is demonstrated with cellfie^8^ that implements the M^2^ DSL [25] so that a modeller can add content in a spreadsheet and cellfie converts that into axioms in the ontology, as demonstrated in Example 7.1 of the textbook. Regarding ODPs (Item D), a content ODP with the current contents is not immediately obvious, but other types of ODPs, such as architectural ones, are easy to illustrate, alike for BioTop [26] but then at the organism-level with an orchestration between foundation, top-domain, and domain-level ontologies, and what are dubbed “exception patterns” in [6] to be used for the deviant cases when remaining within a monotonic logic such as OWL (e.g., penguins as non-flying birds).

## Utility and discussion

The principal utility of the AWO is to be a concrete machine-processable artefact for the related examples and exercises, which we shall turn to first, and subsequently discuss the tutorial ontology.

### Use in exercises and examples

The major utility of the AWO is its use in educational activities for ontology engineering exercises and examples that are described in the “An Introduction to Ontology Engineering" textbook [16]. It is not intended as a real domain ontology, but it is explicitly designed as a tutorial ontology that has a domain ontology flavour to it. Consequently, the subject domain knowledge about African Wildlife has been kept simple, yet amenable to extensions.

**Fig. 2:**
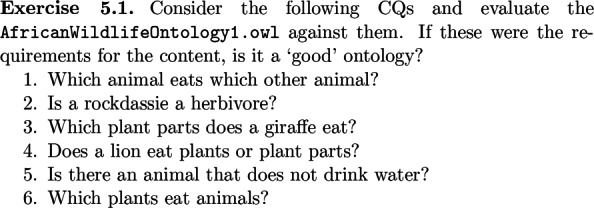
Section of an exercise. Screenshot of the first part of Exercise 5.1 in [16], which lets the student experiment with requirements for the content of an ontology, trying to find that knowledge, and the task of evaluating an ontology on its quality based on its requirements. The high-level notion of a ‘good’ ontology—compared to ‘less good’, ‘bad’, and ‘worse’—has been introduced earlier in the textbook, which has to be recalled and applied here

An example of an exercise is shown in Fig. 2, which fits within the broader scope of sensitising the student to the notion of quality of an ontology, using the vehicle of competency questions that can be used in the validation stage when evaluating whether the ontology meets its stated goals. It also offers a gentle acquaintance with foundational ontologies with some OWL classes that are either easy or fun to categorise or to elicitate lively debate. For instance, impalas die in the process of being eaten by a lion, where both are straightforwardly subclasses of Physical Object in DOLCE [27] or Independent Continuant in BFO [9], and Death is a type of Achievement or Process boundary, respectively. The exercises of aligning AWO to DOLCE is additionally assisted by the D3 decision diagram [28]. Death/dying also provides an entry point to the alternate modelling styles of process-as-relation vs. process-as-class representation options. Another core distinction in modelling styles are data properties vs. a hierarchy of qualities, for which a use case of elephant’s weight in zoos across the world is used (Section 6.1.1 of [16]).

While the emphasis in this paper is on modelling and engineering aspects, the AWO is still suitable for teaching about OWL language features and automated reasoning, as noted before regarding the deductions (e.g., $\textsf {Lion} \sqsubseteq \textsf {Carnivore}$), and use of language features such as transitivity and (ir)reflexivity with parthood. Straightforward examples for demonstrating unsatisfiability are multiple inheritance of Omnivore to the disjoint Carnivore and Herbivore or to set the domain of eats to Animal resulting in an unsatisfiable CarnivorousPlant.

Additional variants of the AWO are in progress, which zoom in on subject domains with corresponding exercises that are not yet covered in the introductory textbook. Among others, a future ‘version 5’ may be the engineering aspects of importing, aligning, and integrating, another domain ontology rather than a foundational ontology, such as a module of the Environment Ontology with the habitat information or a tourism ontology, with a corresponding sample answer file. The former option would be more suitable for ontology development in ecology, whereas the latter is a more practical option in a tutorial/course for people in other disciplines. Other themes that have not been covered explicitly yet but easily can be applied to the AWO are modularisation [29] and Ontology-Based Data Access with its recent tools [30], and it could be assessed against the MIRO guidelines for reporting on ontologies [31].

### Discussion

The AWO meets most of the tutorial ontology requirements that evolved and extended over the years. The AWO goes beyond extant tutorial ontologies that overwhelmingly focus only on demonstrating language features and automated reasoning, or how to use a specific version of a specific tool. In particular, the AWO brings in ontology development aspects, such as competency questions and alignment to a foundational ontology, among others.

The illustrations of gradual quality improvements—common in ontology development—go beyond the notion that a new version only uses more language features, as in Family History [5] and University^9^. In particular, there are improvements on aspects regarding, among others, content, naming, annotations, and foundational ontology alignment.

Also, care has been taken in representing the knowledge, such as avoiding some common pitfalls like the class-as-instance and certain naming issues like ‘and’, ‘or’ or negation in a term [13]. Unlike other tutorial ontologies, including the popular Pizza and Wine, it is richly annotated with informal descriptions, pointers to introductory domain knowledge, and questions for further exploration of a modelling topic.

Tutorial ontology subject domains such as one’s family history, a university, or one’s pets are distinctly focussed on individual application scenarios that may serve database development, but do not give an educationally good flavour of typical scopes of domain ontologies. In that regard, pizzas and wines fare somewhat better, which, however, have repetitive content, such as listing all ingredients of pizza topping. Contrast this with animal wildlife, where it suffices already to represent that a lion eats animals to have it classified automatically as a carnivore. The wildlife subject domain is generic rather than specific for one application scenario, and therewith less predisposed to a myopic ‘my thing only’ thinking that is prevalent when students encounter ontologies for a first time (a regular occurrence at least in the author’s classes, carried over from software design). Last, but not least, besides its international appeal, African wildlife is obviously relevant for South Africa, where the author and most of her students are based, and it fits with the trend to make curricula regionally relevant. This is also reflected in an isiZulu and an Afrikaans version of the ontology and introductory aspects on term use for ontologies in a multilingual setting, as Rockdassie is not a Standard English word yet is widely accepted in South African English. Overall regarding the content of a tutorial ontology, however, it is a balancing act between simplicity and ontological precision and correctness, as [6] also noted, and international and national relevance, as well as an estimation what may be assumed to be general common sense knowledge by novice ontologists.

## Conclusions

The paper introduced the African Wildlife Ontology tutorial ontologies, which is a set of ontologies used for a variety of ontology development examples and exercises. Considering possible desirable educational outcomes, 22 requirements were formulated that a tutorial ontology should meet. The AWO meets most of these requirements, therewith improving over its predecessors especially reading the notions of evolution of ontology quality several ontology development tasks beyond getting the axioms into an OWL file, such as alignment to a foundational ontology and satisfying competency questions.

Both the 22 requirements and the AWO are relevant to the field of ontology engineering in particular, especially for enhancing course material, which, it is hoped, will result in further quality improvements of the actual ontologies that developers are building.

## Abbreviations

AWO: African wildlife ontology
CDM: Conceptual data model
FMA: Foundational model of anatomy
FO: Foundational ontology
ODP: Ontology design pattern
OE: Ontology engineering
OWL: Web ontology language

## Footnotes

1 http://www.w3.org/TR/2003/PR-owl-guide-20031209/wine

2 http://xiphoid.biostr.washington.edu/fma/shirt_ontology/shirt_ontology_1.php

3 http://www.workingontologist.org/Examples.zip; Last accessed: 26-11-2018.

4 Recent political issues include complaints with subject domains of exercises that perpetuate stereotypes and simplifications, such as, but not limited to, the gender binary, who can marry whom, and gendered subject domains perceived to reside at the edges of the spectrum.

5 *in casu*, declaring eats too restrictively with as domain only Animal: then either it will result in an unsatisfiable CarnivorousPlant (if Animal and Plant are declared disjoint) or it will result in the undesirable deduction that CarnivorousPlant$\sqsubseteq $Animal

6 https://www.kaggle.com/cites/cites-wildlife-trade-database

7 http://www.tdwg.org/

8 https://github.com/protegeproject/cellfie-plugin

9 http://owl.man.ac.uk/2005/07/sssw/university.html

## References

[1] Schulz S, Boeker M, Vera-Ramos JA, Jansen L, Borgo S, Kutz O (2017). Pizza & wine: The need for educational tools for foundational ontologies. Proceedings of the Joint Ontology Workshops 2017 (JOWO’17), CEUR-WS, vol. 2050.

[2] Noy NF, McGuinness DL. Ontology development 101: A guide to creating your first ontology. Technical Report KSL-01-05, and Stanford Medical Informatics Technical Report SMI-2001-0880, Stanford Knowledge Systems Laboratory. 2001.

[3] Smith MK, Welty C, McGuinness DL. OWL Web Ontology Language guide. W3c recommendation, W3C. 2004. http://www.w3.org/TR/owl-guide/. Accessed 17 June 2020.

[4] Rector A, Drummond N, Horridge M, Rogers L, Knublauch H, Stevens R, Wang H, Wroe Csallner C (2004). OWL pizzas: Practical experience of teaching OWL-DL: Common errors & common patterns. Proceedings of the 14th International Conference Knowledge Acquisition, Modeling and Management (EKAW’04), LNCS, vol. 3257.

[5] Stevens R, Stevens M, Matentzoglu N, Jupp S (2013). Manchester Family History Advanced OWL Tutorial.

[6] Schober D, Grewe N, Röhl J, Boeker M, Boeker M, Herre H, Hoehndorf R, Loebe F (2012). Zooanimals.owl: A didactically sound example-ontology for teaching description logics in OWL 2. OBML 2012 Workshop Proceedings (OBML’12), IMISE-REPORT.

[7] Allemang D, Hendler J (2008). Semantic Web for the Working Ontologist.

[8] Antoniou G, van Harmelen F (2003). A Semantic Web Primer.

[9] Arp R, Smith B, Spear AD (2015). Building Ontologies with Basic Formal Ontology.

[10] Hitzler P, Krtzsch M, Rudolph S (2009). Foundations of Semantic Web Technologies.

[11] Suárez-Figueroa MC, Gómez-Pérez A, Motta E, Gangemi A (2012). Ontology Engineering in a Networked World.

[12] Duque-Ramos A, Fernández-Breis JT, Iniesta M, Dumontier M, Egana Aranguren M, Schulz S, Aussenac-Gilles N, Stevens R (2013). Evaluation of the oquare framework for ontology quality. Expert Syst Appl.

[13] Keet CM, Suárez-Figueroa MC, Poveda-Villalón M, Fred A, Dietz JLG, Liu K, Filipe J (2015). Pitfalls in ontologies and tips to prevent them. Knowledge Discovery, Knowledge Engineering and Knowledge Management: IC3K 2013 Selected Papers, CCIS, vol. 454.

[14] Poveda-Villalón M, Suárez-Figueroa MC, Gómez-Pérez A, ten Teije A (2012). Validating ontologies with OOPS!. 18th International Conference on Knowledge Engineering and Knowledge Management (EKAW’12), LNAI, vol. 7603.

[15] Schulz S, Seddig-Raufie D, Grewe N, Röhl J, Schober D, Boeker M, Jansen L. Guideline on developing good ontologies in the biomedical domain with description logics. Technocal report (December 2012). v1.0. http://www.purl.org/goodod/guideline. Accessed 17 June 2020.

[16] Keet CM (2018). An Introduction to Ontology Engineering. Computing, vol. 20.

[17] Uschold M, Gruninger M (1996). Ontologies: principles, methods and applications. Knowl Eng Rev.

[18] Wisniewski D, Potoniec J, Lawrynowicz A, Keet CM. Competency questions and SPARQL-OWL queries dataset and analysis. Technical Report 1811.09529. 2018. https://arxiv.org/abs/1811.09529.

[19] Motik B, Patel-Schneider PF, Parsia B. OWL 2 web ontology language structural specification and functional-style syntax. W3c recommendation, W3C. 2009. http://www.w3.org/TR/owl2-syntax/. Accessed 17 June 2020.

[20] Distributed Ontology, Model, and Specification Language. Object Management Group. http://www.omg.org/spec/DOL/. Accessed 17 June 2020.

[21] Keet CM, Lawrynowicz A, Sack H (2016). Test-driven development of ontologies. Proceedings of the 13th Extended Semantic Web Conference (ESWC’16). LNCS, vol. 9678.

[22] Garijo D, d’Amato C (2017). WIDOCO: a wizard for documenting ontologies. The Semantic Web – ISWC 2017. LNCS, vol. 10588.

[23] Lohmann S, Link V, Marbach E, Negru S (2015). WebVOWL: Web-based visualization of ontologies. Proceedings of EKAW 2014 Satellite Events. LNAI, vol. 8982.

[24] Buttigieg PL, Morrison N, Smith B, Mungall CJ, Lewis SE (2013). The environment ontology: contextualising biological and biomedical entities. J Biomed Semant.

[25] O’Connor MJ, Halaschek-Wiener C, Musen MA, Patel-Schneider PF (2010). Mapping master: A flexible approach for mapping spreadsheets to OWL. Proceedings of the International Semantic Web Conference 2010 (ISWC’10). LNCS, vol. 6497.

[26] Beisswanger E, Schulz S, Stenzhorn H, Hahn U (2008). BioTop: An upper domain ontology for the life sciences - a description of its current structure, contents, and interfaces to OBO ontologies. Appl Ontol.

[27] Masolo C, Borgo S, Gangemi A, Guarino N, Oltramari A. Ontology Library. WonderWeb Deliverable D18 (ver. 1.0, 31-12-2003). 2003. http://wonderweb.semanticweb.org. Accessed 23 Feb 2005.

[28] Keet CM, Khan MT, Ghidini C (2013). Ontology authoring with FORZA. Proceedings of the 22nd ACM International Conference on Conference on Information & Knowledge Management (CIKM’13).

[29] Khan ZC, Keet CM (2015). An empirically-based framework for ontology modularization. Appl Ontol.

[30] Calvanese D, Cogrel B, Komla-Ebri S, Kontchakov R, Lanti D, Rezk M, Rodriguez-Muro M, Xiao G (2017). Ontop: Answering SPARQL queries over relational databases. Semant Web J.

[31] Matentzoglu N, Vigo M, Jay C, Stevens R (2018). Inference inspector: Improving the verification of ontology authoring actions. Web Semant Sci Serv Agents World Wide Web.

